# Micro-structure and morphology of tailings sand under different oxidation and acidification degree

**DOI:** 10.1038/s41598-022-26130-0

**Published:** 2023-01-18

**Authors:** Gang Wang, Xiqi Liu, Leibo Song, Xiaoming Ma, Wenzhao Chen, Jiaxing Qiao

**Affiliations:** 1 Collaborative Innovation Center for Prevention and Control of Mountain Geological Hazards of Zhejiang Province, Shaoxing University, Shaoxing, 312000 China; 2 Huahui Engineering Design Group Co., Ltd, Shaoxing, 312000 China; 3 School of Civil Engineering, University of South China, Hengyang, 421000 China

**Keywords:** Natural hazards, Engineering, Civil engineering

## Abstract

The tailings pond is a dangerous source of man-made debris flow with high potential energy. The oxidative acidification of tailings may cause the instability of the pond and induce serious safety accidents. The influence of oxidation and acidification degree on macro mechanical properties of tailings is discussed from the aspects of mineral composition and microstructure. The results show that as the degree of oxidation and acidification of tailings sand increases, the overall structural performance and load-bearing capacity decrease, and its cohesion (*c*) and internal friction angle (*φ*) show a decreasing trend. In fact, the engineering properties of tailings with different oxidation and acidification degrees are dominated by the physicochemical composition and structural characteristics. On the one hand, as the degree of oxidation increases, acidic substance will neutralize with CaCO_3_ and CaMg(CO_3_)_2_, resulting in the loss of cemented substance and the decrease of cementation force between tailing sand particles as well as the gradual destruction of the integrity of tailing sand. On the other hand, the increase of oxidation and acidification degree of tailing sand leads to a gradual reduction of outline (2*D*) fractal dimension and gray surface (3*D*) fractal dimension of surface laminated structure as well as the obvious reduction of laminated structure and its roughness of tailings sand.

## Introduction

A large number of tailings and industrial waste residues produced by mineral resources mining are stored in tailings pond except for a small part of construction materials^1–6^. Therefore, as a major mining producer, China has built tens of thousands of tailings ponds. The acid wastewater produced by tailings acidification not only causes serious pollution to the ecological environment downstream of the mine, but also has a strong corrosive effect on the tailings dam body, resulting in the loosening of the dam body structure, and even dam failure accidents. The high potential tailings debris flow generated by dam failure accidents will cause great loss of life and property of downstream residents.

Domestic and foreign scholars have carried out a lot of experimental research on the mechanical properties of tailings sand. Peng et al.^7^ took the damming tailings of Xiaoda’e tailings pond as the research object, and carried out a comparative study of dynamic triaxial tests on two kinds of tailings, namely tailing silt sand and tailing silt soil. The results showed that the dynamic strength and dynamic elastic modulus of tailing silt soil were significantly lower than those of tailing silt sand. After mechanical compaction, the dynamic characteristics of the two tailings were improved, and the improvement effect of tailings silt soil was more significant. Qiao et al.^8^ analyzed the influence of fine particle content on the engineering properties of tailings. The results showed that due to the influence of the particle composition of the original tailings, the cohesion of the tailings increased gradually with the increase of the fine particle content, while the relative change of the internal friction angle was small. The permeability coefficient was greatly affected by the fine particle content, and decreased rapidly with the increase of the content. Wu et al.^9^ studied the influence of powder content on the mechanical properties of tailings and its mesoscopic mechanism. The results showed that the smaller the particle size and elastic modulus of the powder were, the larger the soil deformation under the same force was. From the mesoscopic point of view, the increase of powder content weakened the original strength chain, which was an important reason for the powder content effect. Grebby et al.^10^ used the intermittent small baseline subset technique of satellite interferometric synthetic aperture radar data to evaluate the tailings dam break disaster. According to the observed precursor deformation, the time of dam collapse could be predicted. Satellite-based monitoring technology may help reduce similar disasters in the future. On the basis of laboratory tests, Lolaev et al.^11^ proposed a method to determine the filtration consolidation and secondary consolidation coefficients of alluvium tailings sand according to physical conditions, density and water saturation, and established a mathematical model to calculate the consolidation time of tailings sand. Jin et al.^12,13^ studied the acceleration, pore pressure, earth pressure and displacement of the dam under the action of different PGA peak acceleration seismic waves through shaking table tests. The results showed that the acceleration, pore pressure, earth pressure and displacement of the dam decreased with the increase of peak acceleration. The free surface of the dam was more prone to liquefaction, and the pore pressure of the dam presented three stages of rapid increase, sharp decrease and slow dissipation over time. Chen et al.^14^ conducted consolidated drainage (CD) triaxial compression test by using high-pressure triaxial apparatus, studied the strength and deformation characteristics of tailings, deduced the constitutive relationship of tailings under high confining pressure, and carried out qualitative and quantitative analysis of tailings particle breakage. Ke et al.^15^ conducted a series of monotonic cyclic triaxial undrained tests on low plastic fine tailings (Taiping tailings) to study their mechanical response. The unified viscous energy dissipation ratio (VEDR) was used to establish the energy-based cyclic loading failure mode research method. The VEDR failure criterion with the peak value of the VEDR as the fault point was proposed. Rui et al.^16^ found that anisotropy has a great influence on the peak shear strength of tailing sand, which is mainly due to the influence of anisotropy on soil dilatancy. Fourie et al.^17^ conducted triaxial undrained compression tests on unsaturated tailings sand samples, and the results showed that under undrained loading, the liquefaction capacity of tailing sand would be reduced due to the emergence of bubbles in the pores. Jin et al.^12,13^ used shaking table to study the flow characteristics of tailings after liquefaction. The results showed that the apparent viscosity of tailings after liquefaction decreased first and then increased with the increase of strain rate, and the tailings sand after liquefaction was a fluid model.

Most of the above researches focus on the macroscopic mechanical properties of tailings sand, but the internal structure of natural rock and soil generally exists uneven structural characteristics, which determines the internal stress and strain distribution of such materials under external load. The change of its macroscopic mechanical properties has a great relationship with the change of its micro-structure^18–21^. In addition, there are few studies on the physical properties of the fine microscopic particle structure of tailings sand at present, and there is no reliable theory that can quantitatively explain the mechanism of complex particles affecting the physical properties of soil. There is still a lot of research space in the establishment and verification of mathematical models. In this regard, pyrite, the main sulfide mineral of tailings acidification and oxidation, is used in this paper. Based on the research methods of soil science and soil mechanics, the evolution law of pyrite tailings sand with different degrees of oxidation and acidification was expounded from the aspects of mineral composition and microstructure. This study has certain theoretical significance and engineering guidance value for understanding the disaster mechanism and safety protection of tailings dam.

## Materials and methods

The tailings sand studied in this paper was from a lead–zinc tailings dam in Hunan Province, China. To ensure that the samples obtained were representative, ten sampling sites were set up on the beach along the direction perpendicular to the dam axis. After stripping 20 cm of surface soil at each sampling site, the corresponding sampling work was carried out. Under natural conditions, the oxidation of pyrite is controlled by surface reaction, which is mainly determined by chemical reaction conditions (H_2_O and O_2_). At the same time, it also determines that the weathering process of pyrite under natural conditions is long. The sulfides in the materials taken in this paper are mainly pyrite, with a very small amount of arsenopyrite. Therefore, this paper mainly considers the oxidation of pyrite produces acid. The oxidation of pyrite under natural conditions is an acid production process, and the final products are Fe^3+^ and SO_4_^2−^. Due to the slow reaction under natural conditions, relevant studies show that H_2_O_2_ can accelerate the oxidation process of pyrite^22^. Therefore, in order to speed up the progress of the test, this paper uses H_2_O_2_ to oxidize pyrite under acidic conditions. The oxidation reaction equation is as follows.

1 $$2{\text{FeS}}_{2} + 15{\text{H}}_{2} {\text{O}}_{2} \to {\text{Fe}}_{2} ({\text{SO}}_{4} )_{3} + {\text{H}}_{2} {\text{SO}}_{4} + 14{\text{H}}_{2} {\text{O}}$$

It can be seen from the above formula that the final products of pyrite oxidized by H_2_O_2_ in acidic solution are also Fe^3+^ and SO_4_^2−^, which are consistent with the oxidation products under natural conditions.

In order to simulate the oxidation process of tailings in natural environment under laboratory conditions, hydrogen peroxide with density of 1.11 g/mol and mass fraction of 30%, concentrated sulfuric acid with density of 1.84 g/mol and mass fraction of 98% are selected to perform soaking tests on the tailings with different degrees of oxidation. The specifications of the drugs used are analytical pure solutions. The amount of H_2_O_2_ under different oxidation degrees is calculated according to the oxidation degree of pyrite.

2 $$V_{n} = \frac{{W_{n} \times 2250 \times 15 \times 34}}{30\% \times 1.11 \times 240}$$

where *V*_*n*_ represents the amount of H_2_O_2_ under different oxidation degrees (*n* = 1, 2, 3, 4), and *W*_*n*_ represents the oxidation degree of pyrite. In this paper, four oxidation conditions are selected as *W*_1_ = 25%, *W*_2_ = 50%, *W*_3_ = 75%, and *W*_4_ = 100%. The test scheme is shown in Table 1, aiming to study the change law of physical properties of tailings after different oxidation degrees.

**Table 1:** Test scheme.

Oxidation degree/%	Tailings sand /kg	H_2_O_2_/L	H_2_SO_4_/L	Oxidation time/h
0	15	0	5	96
25	15	0.4	5	96
50	15	0.8	5	96
75	15	1.2	5	96
100	15	1.6	5	96

The mineral composition of different oxidation degrees was analyzed by MLA (Mineral Liberation Analyser), and the results are shown in Table 2, where only the mineral composition that produced the oxidation acidification reaction was listed.

**Table 2:** Mineral composition of tailings sand samples under different oxidation degrees (mass%).

Group number	Theoretical oxidation degree/%	Actual oxidation degree/%	Mineral composition				
			SiO_2_	FeS_2_	CaCO_3_	CaMg (CO_3_)_2_	FeAlS
I	0	0	64.60	15.80	9.30	3.70	0.95
II	25	23	65.84	12.17	7.52	3.50	0.76
III	50	46	66.53	8.53	4.43	2.87	0.57
IV	75	71	67.68	4.58	1.23	2.21	0.29
V	100	92	68.42	1.21	0	1.94	0.09

Through actual measurement, the mass percentage of pyrite is obtained, and the oxidation degree and theoretical oxidation degree are calculated to evaluate the oxidation effect of pyrite in laboratory test. The calculation formula of pyrite oxidation degree *X* is as follows.

3 $$X = \frac{{M_{1} - M_{2} }}{{M_{1} }} \times 100\%$$

where *M*_1_ is the mass percentage of pyrite (FeS_2_) in original tailings sand and M_2_ is the mass percentage of pyrite in oxidized tailings sand.

It can be seen from Table 2 that the actual oxidation degree of pyrite is generally lower than the theoretical oxidation degree, indicating that pyrite in tailings sand is not fully oxidized. The main reason is that there is a small amount of decomposition loss of H_2_O_2_, but the theoretical oxidation degree is not much different from the actual oxidation degree, which can be approximately expressed as the theoretical oxidation degree.

**Figure 1:**
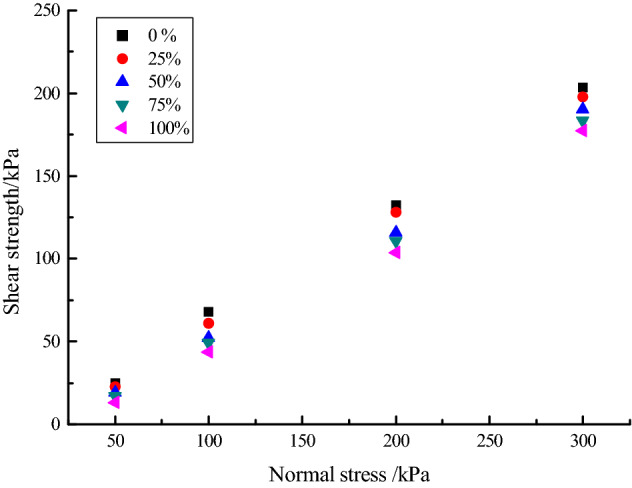
Relationship between shear strength and normal stress of tailings under different oxidation degrees.

Direct shear tests under different oxidation degrees were conducted on saturated tailings samples with a compactness of 85%. TZJ-4 automatic quadruple electric direct shear apparatus was used in the experiment. The vertical pressure was set at four levels, namely 50 kPa, 100 kPa, 200 kPa, 300 kPa and a total of 20 groups of shear tests were conducted. The direct shear test was carried out at the loading rate of 0.8 mm/min^23–25^. The experimental results are shown in Fig. 1.

## Results and discussion

### Analysis of shear degradation characteristics of tailings sand

According to the relationship between shear stress and vertical pressure in Fig. 1, the cohesion *c* and internal friction angle *φ* of tailings sand under different oxidation degrees are obtained as shown in Table 3.

**Table 3:** Changes of mechanical parameters of tailings sand under different oxidation degrees.

Material parameters	Oxidation and acidification degree %				
	0	25	50	75	100
*c*/kPa	6.8	5.6	5.16	4.2	2.68
*φ*/°	31.8	31.3	31.2	30.5	29.5

It can be seen from Table 3 that the peak strength matches the residual strength. As the degree of oxidation and acidification of the tailings sand increases, the cohesion (c) and internal friction angle (*φ*) will decrease, which is similar to the law found by many scholars^26,27^. The shear resistance of particle materials is related to factors such as cementation properties and friction between particles. The cohesion is affected by the cementing material on the particle surface, and the internal friction angle and the friction between particles change with the particle roughness. The above analysis shows that the oxidation and acidification of tailings sand reduce the overall mechanical properties of tailings sand. The changes of mechanical properties of tailings sand under different oxidation and acidification degrees are related to the changes of mineral composition and physical properties of micro-structure of tailings sand surface after acidification.

Macroscopic failure phenomenon of materials is a comprehensive manifestation of many microscopic fractures^28–30^. In order to observe the morphological characteristics of tailings sand under different oxidation and acidification degrees from the microscopic point of view, ZEISS- Gemini-SEM 300 field emission scanning electron microscope was used to analyze the micro-structure and mineral composition of tailings sand particles under different oxidation and acidification degrees. Before the scanning electron microscope image acquisition, it is necessary to conduct gold plating on the sample surface, and then conduct microscopic observation to collect secondary electronic images.

### Analysis of the microscopic morphology of tailings sand particles

**Figure 2:**
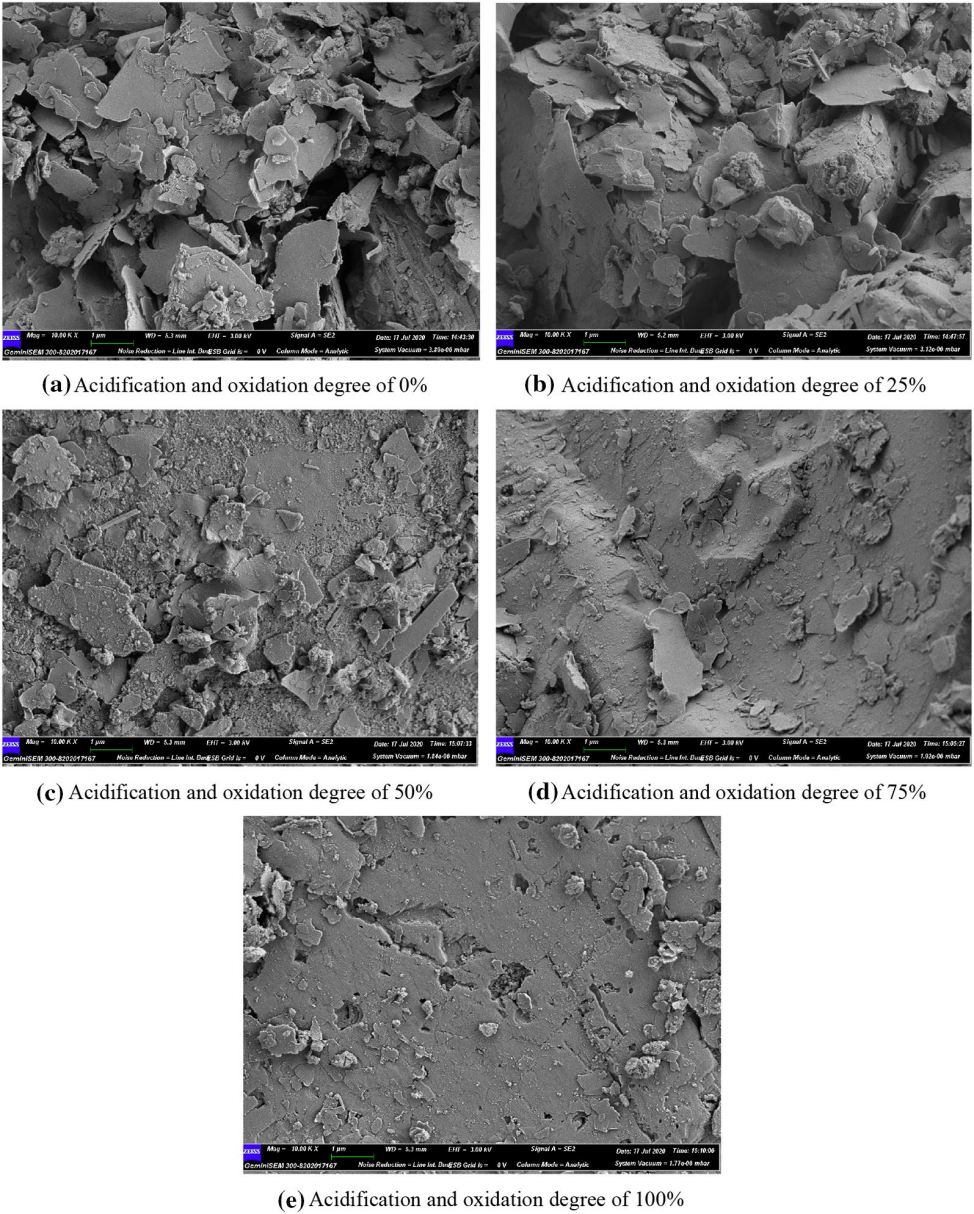
SEM photos of tailings sand under different oxidation and acidification degrees.

Figure 2 shows the SEM photos of tailings sand under different oxidation and acidification degrees. It can be seen that the surface structure of tailings sand can be clearly seen when the magnification of tailings sand particle surface is × 10,000 times. The surface of tailings sand with low oxidation and acidification degrees (0%, 25%) has obvious interlaced laminated structure, and mineral laminated structure is the basic structural unit of tailings sand surface under low oxidation and acidification degrees. The laminated structure forms a loose matrix with weak cementation on the surface of tailings sand, and the cementing material envelops the skeleton particles of tailings. As the degree of oxidation and acidification increases (50%, 75%, 100%), it can be seen from the scanning electron microscope images that the lamination structure on the surface of the tailings sand is significantly reduced, and the microscopic surface is compact and flat. It indicates that the degree of oxidation and acidification will affect the laminated structure of the micro-surface of tailings sand, and then change its macroscopic mechanical properties. The changes of the macroscopic cohesion and internal friction angle of tailings sand under different oxidative and acidification degrees are inherently related to the micro-structure evolution of the samples.

### Mineral composition analysis of tailings sand

**Figure 3:**
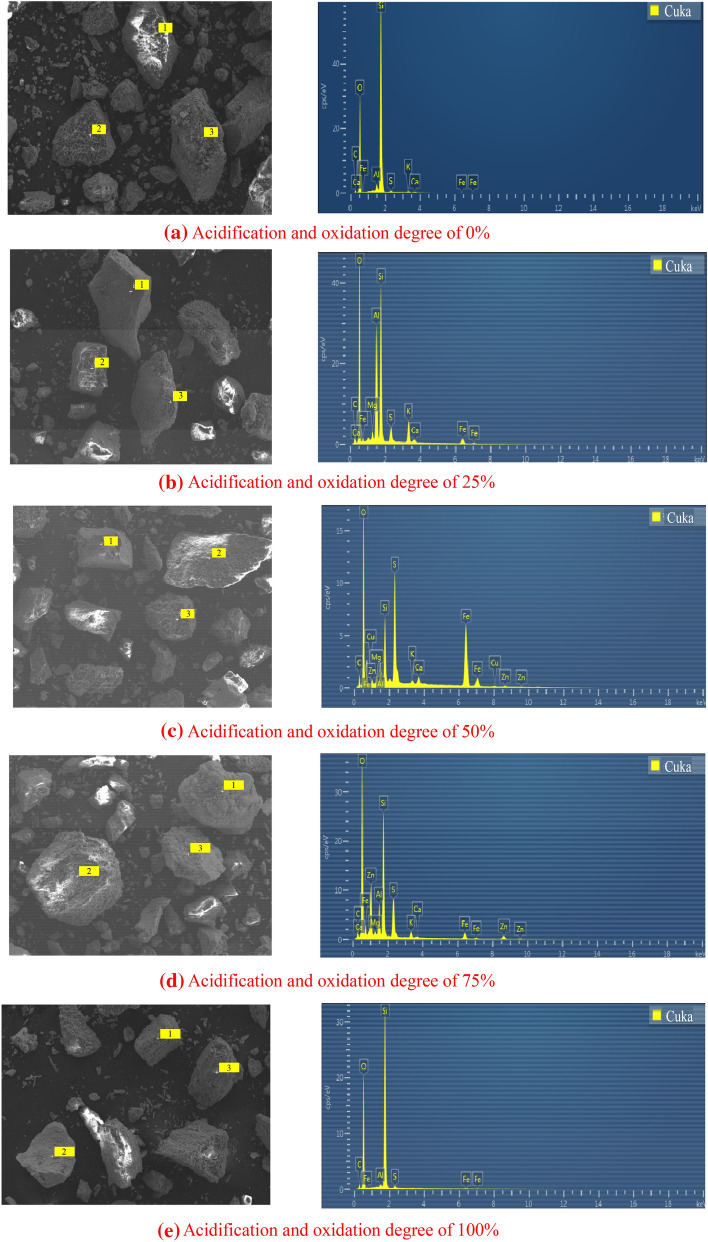
The EDS test point domain and element spectrogram of tailings sand particles with different oxidation and acidification degree.

In order to analyze the laminated structure of tailings sand surface, five kinds of tailings sand with different acidification and oxidation degrees were selected to perform conventional EDS analysis on their micro-surface components, as shown in Fig. 3 and Table 4.

**Table 4:** Energy spectrum analysis of tailings sand under different acidification and oxidation degree.

Element	Oxidation degree 0%		Oxidation degree 25%		Oxidation degree 50%		Oxidation degree 75%		Oxidation degree 100%	
	Mass ratio	Atomic ratio	Mass ratio	Atomic ratio	Mass ratio	Atomic ratio	Mass ratio	Atomic ratio	Mass ratio	Atomic ratio
O	25.51	28.67	25.13	42.47	40.49	52.52	45.19	55.76	55.73	63.93
Si	3.07	2.51	5.93	5.71	15.76	11.65	33.55	21.92	44.80	31.49
AI	4.00	3.07	1.94	1.95	1.19	0.87	1.15	0.91	0.41	0.28
Fe	28.21	10.78	21.90	10.60	5.77	2.14	1.18	0.42	1.06	0.35
Mg	1.44	0.77	0.61	0.42	0.41	0.21	0.30	0.16	0.13	0.06
Ca	12.40	8.40	6.20	4.20	2.60	1.40	2.30	1.20	1.90	1.00
C	24.01	42.64	12.16	21.01	8.66	13.23	7.14	16.08	6.65	10.92
S	20.93	13.93	11.8	9.95	6.33	4.10	0.32	0.2	0.32	0.2

Figure 3 shows the EDS test results of tailings sand under different acidification and oxidation degrees. The EDS test and SEM test are carried out simultaneously. The EDS test position is the typical structure morphology observed in the process of SEM test. The EDS test is mainly carried out in point domain. Table 4 shows the EDS analysis results of tailings sand under different acidification and oxidation degrees. The mass percentage and atomic percentage of elements in each test point domain are analyzed in detail. It can be seen from the table that the main chemical elements in tailings sand are O, Si, AI, Fe, C, Ca and other elements. Except for the element Fe, the contents of O, C and Ca are the highest in the test point domain of tailings sand without acidification and oxidation, and its mass percentage and atomic percentage are more than 8%. It can be inferred that the cements between particles are CaCO_3_ (calcite) and CaMg(CO_3_)_2_. The atomic ratio of Ca/Si in the point domain of unacidified tailings sand is 3.35. With the increase of acidification and oxidation degree, the content of Ca is much higher than that of Si. The possible reasons are as follows. The cementing material wraps the tailings sand particles but fails to detect the Si element, and the cementing material contains more O, C and Ca elements. With the increase of oxidation and acidification degree of tailings sand, the contents of Si, O and other elements increase obviously, and the atomic ratios of Ca/Si in the point domain are 0.74, 0.12, 0.05, 0.03, respectively, which decreases gradually. The main reason is that the oxidation of pyrite produces sulfuric acid, which neutralizes with CaCO_3_ and CaMg (CO_3_)_2_, and the cementing material on the surface of tailings sand is lost.

The EDS analysis shows that with the increase of oxidation and acidification degree, the mass percentages of Ca in the inter-granular crystals are 8.4%, 4.2%, 1.4%, 1.2%, 1.00%, respectively, and the mass percentages of C are 42.64%, 21.01%, 13.23%, 16.08%, 10.92%, respectively. The mass percentage of Ca and C shows a decreasing trend. These crystals are distributed in the gap of the tailings sand particles, and the loose particles are consolidated into a whole through their own adhesive force, acting as the “skeleton” of the tailings sand, that is, as a kind of cementing material to strengthen the connection of the tailings sand particles, and improve the overall structural performance and bearing capacity of the tailings sand. The surface of the tailings sand particles with a lower degree of oxidation has more mineral components, and the particle size is larger. The larger the tailings sand particles are, the rougher the particle surface is, and the greater the surface friction is. At the same time, the larger the tailings sand particles are, the greater the bite force generated by the particle linkage is, and the greater the internal friction angle is. In addition, the tailings sand particles with lower oxidation degree has more cements, mainly distributed at the particle connection, which results that the tailings sand with lower oxidation degree has higher cementation force (cohesion). The increase of oxidation and acidification degree leads to the decrease of cementation force, the expansion of cracks, the gradual destruction of aggregates, and the obvious decrease of internal friction angle and cohesion of tailings sand.

### Fractal analysis of micro-fracture surface

The image by SEM is gray image, in which the gray level of black is 0, and the gray level of white is 255. In the SEM micro-fracture image of tailings sand in this paper, the light color part near white usually shows high crystal fluctuation, and the dark color part near black is pore or low fluctuation area. The irregularity of tailings sand surface can be quantitatively described by fractal dimension. Therefore, in order to further analyze the surface roughness and distribution of surface polymers (laminated structure) of the five kinds of oxidized and acidified tailings sand, according to different gray values, the two-dimensional crystal edge map and three-dimensional structure surface space surface map of the microscopic surface of tailings sand under typical different degrees of oxidation and acidification can be established by MATLAB.

Based on the numericalization of the microscopic surface of tailings sand, the profile lines are extracted from the surface of tailings sand to numerically express the surface roughness shape. In this paper, the fractal research method of two kinds of calculating methods of fractal dimensionis selected to explore the influence of acidification and oxidation on the structural distribution (2*D*) and surface flatness (3*D*) of the surface lamination of tailings sand. The 2*D* fractal dimension is mainly related to cohesion, while the 3*D* fractal dimension is mainly related to internal friction angle.

**Figure 4:**
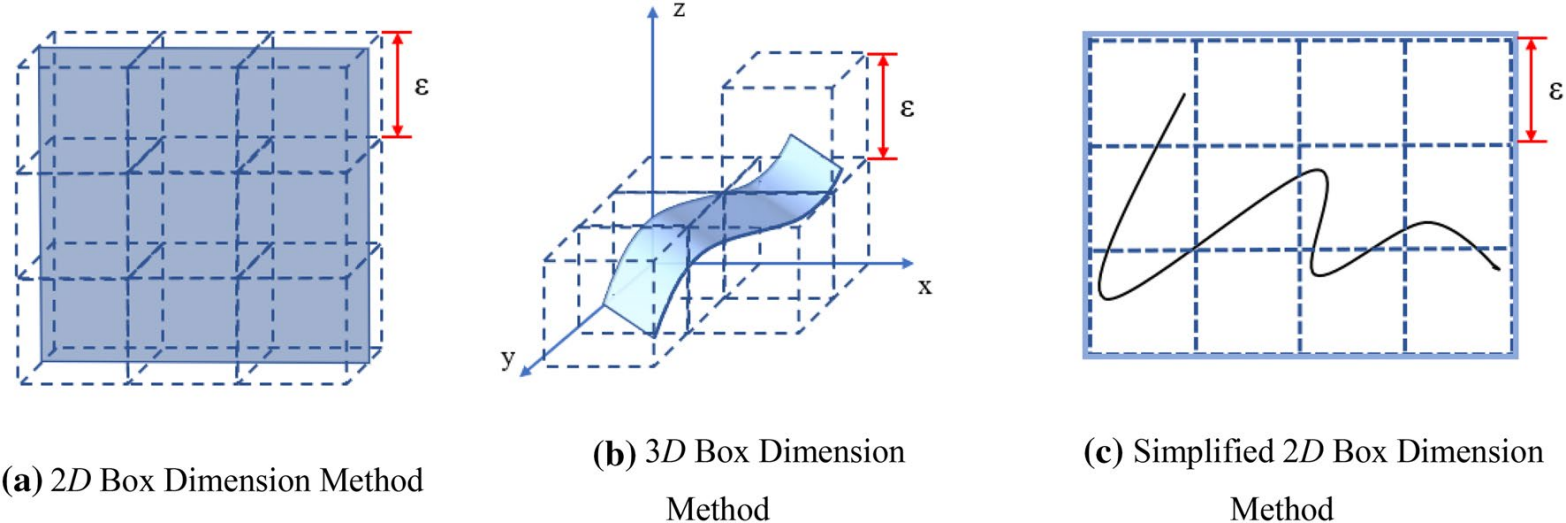
Schematic diagram of box dimension method.

Fractal dimension is an important parameter to describe fractal characteristics. For different research objects, different fractal methods should be adopted. The commonly used methods for calculating the fractal dimension of material surface include perimeter-area relationship method, power law spectrum method, self-affine fractal method, box dimension method and Hausdroff dimension method and so on^31^. Among them, box dimension method is more intuitive, practical, widely used and easy to program. Therefore, the fractal dimension of two-dimensional and three-dimensional images in this paper is calculated by using the fractal principle of box dimension, as shown in Fig. 4.

If the cube box with side length of ε is used to measure the measured object, some of the boxes are covered with the image, and some of the boxes are empty. The number of boxes covered with image is denoted as *N*(*ε*). Then reduce the side length of the box to *ε*_i_ to get the corresponding *N*(*ε*_*i*_), and the relationship can be obtained as follows.

4 $$N(\varepsilon_{i} ) \propto \varepsilon_{i}^{ - D}$$

where *D* is a dimension.

When *ε* tends to 0, the fractal dimension *D* can be obtained:

5 $$D = \mathop {\lim }\limits_{\varepsilon \to 0} \frac{\log N(\varepsilon )}{{ - \log (\varepsilon )}}$$

In the actual calculation process, only the limited ε value can be taken to calculate a series of *N*(ε_i_) and *ε*_i_. Then the numerical values are fitted by the least square method in the double logarithmic coordinate system, and the slope of the line is fractal dimension *D*. Generally, the 2*D* fractal dimension is between 1–2 and the 3*D* fractal dimension is between 2 and 3. In order to simplify the calculation, the side length of the box in MATLAB is taken as the integer power of 2 pixels (px), and the size of the image of the research object is 512 px × 512 px.

After the image of the fracture surface is obtained by SEM, the analysis field in the image is extracted, processed and analyzed. Some regions of the image with magnification of 10,000 are extracted as the research objects. Each numbered image needs to extract three distinct regions with clear and representative crystals. After numbering, two-dimensional and three-dimensional image fractals are performed respectively.

**Figure 5:**
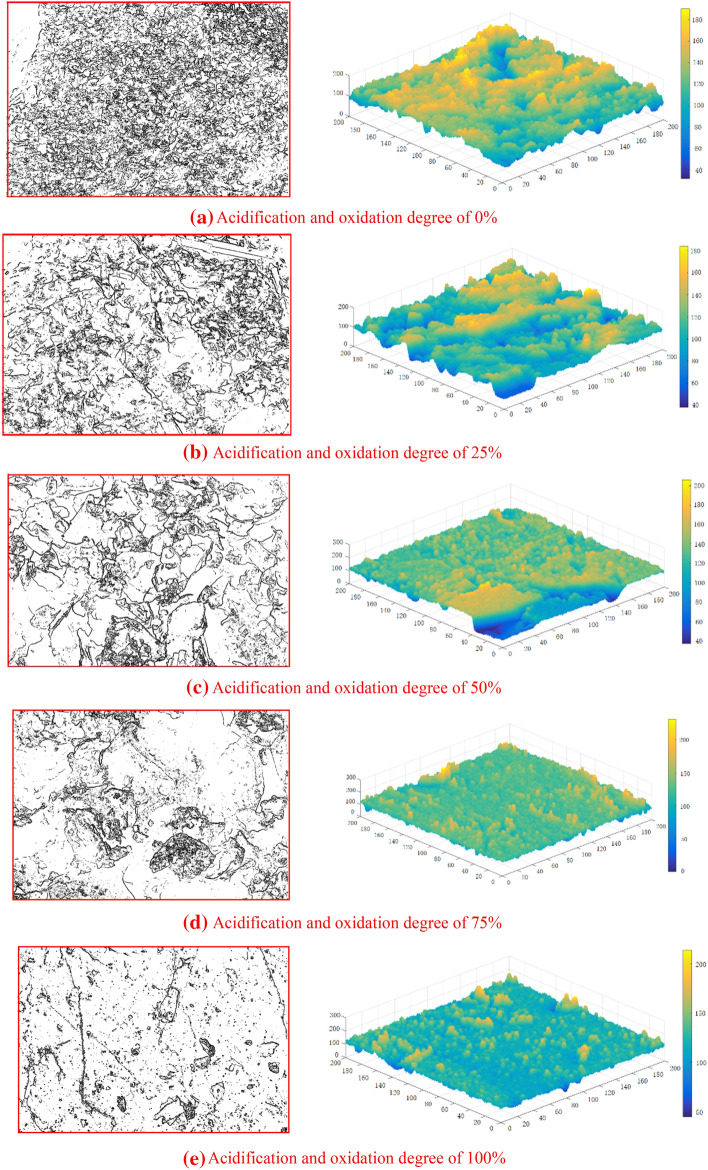
The Binary image and Microscopic 3*D* structure of micro-fracture surfaces of tailing sand under different oxidation and acidification degrees.

As shown in Fig. 5, for the two-dimensional image, the box can be simplified from the cube to a square for the convenience of calculation. The crystal contour from the numbered image can be extracted by using MATLAB and binarization processing can be performed on it. For three-dimensional images, according to the gray value of the graphics, the microscopic three-dimensional structures corresponding to different micro-fracture surfaces can be established by MATLAB^32,33^.

The 2*D* fractal dimension of the crystal contour of the surface image in this region can be obtained by using MATLAB to extract the crystal contour from the numbered image and binarize it. Based on the numerical values of the micro-surface roughness shape of debris, the corresponding 3*D* fractal dimension can be obtained through the geometric information of the gray surface. Three positions of tailings sand particles with different oxidation degrees are randomly selected for calculation, and the average value is taken.The results are shown in Table 5.

**Table 5:** Calculation results of 2*D* and 3*D* fractal dimensions of micro-surface.

Category	Oxidation degree 0%		Oxidation degree 25%		Oxidation degree 50%		Oxidation degree 75%		Oxidation degree 100%	
	2*D*	3*D*	2*D*	3*D*	2*D*	3*D*	2*D*	3*D*	2*D*	3*D*
Fractal dimension	1.754	2.302	1.741	2.277	1.709	2.231	1.576	2.223	1.440	2.099
	1.765	2.310	1.747	2.268	1.682	2.251	1.623	2.229	1.504	2.109
	1.734	2.311	1.724	2.286	1.712	2.250	1.625	2.230	1.559	2.117
Average	1.751	2.308	1.737	2.277	1.701	2.244	1.608	2.227	1.501	2.108

**Figure 6:**
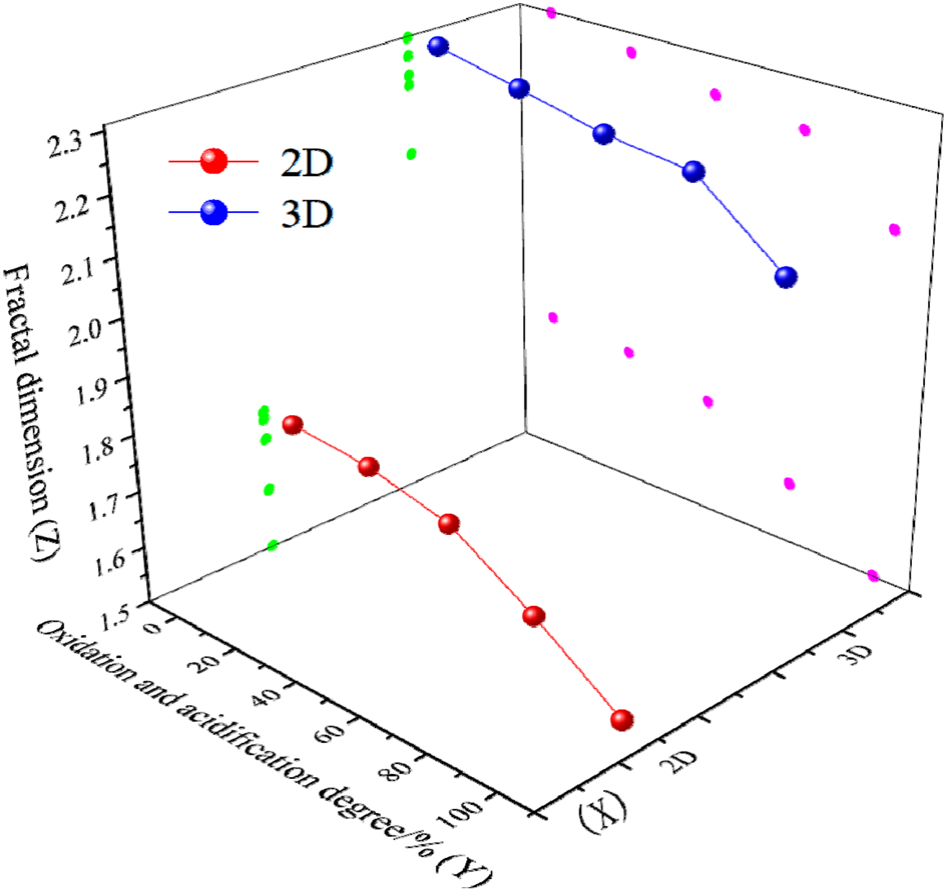
Data statistical charts of oxidation and acidification degree, dimension and fractal dimension.

It can be seen from Table 5 that with the increase of oxidation and acidification degree of tailings sand, the (2*D*) fractal dimension of the laminated structure contour of the tailings sand surface and the (3*D*) fractal dimension of the gray surface gradually decrease. Based on this, the data statistical chart of oxidation and acidification degree, dimension and fractal dimension is established. As shown in Fig. 6, the blue and red sphere is the fractal dimension, and the green and pink circles are the projections of the fractal dimension on the XZ and YZ planes.

It can be seen from Fig. 6 that there is no intersection between 2 and 3*D* fractal dimensions. the 2*D* fractal dimension of the laminated structure contour represents the arrangement of laminated structure. The smaller the fractal dimension is, the smoother the surface of tailings sand particles is, and the sparser the distribution of the laminated structure is. The 3*D* fractal dimension of the gray surface represents the roughness of tailings sand surface. The smaller the fractal dimension is, the smoother the surface is. In conclusion, with the increase of the degree of oxidation and acidification of tailings sand, the laminated structure and the roughness of tailings sand particle surface decrease. However, 2*D* fractal dimension is more sensitive to the change of oxidation and acidification, indicating that the cohesion is greatly affected by oxidation and acidification.

### Relationship between oxidation-acidification degree and fracture surface morphology

The experimental results show that the oxidation and acidification degree of tailings sand is inversely proportional to the structural arrangement density and surface flatness of micro-surface laminated structure. But the influence weight of oxidation and acidification degree on the two factors can not be determined. This paper attempts to determine the statistical relationship among the three by statistical methods. In order to facilitate the calculation, the average fractal dimension of each numbered sample is selected for statistics. In order to eliminate the influence of different units on the analysis results, the data were standardized. The formula is as follows:

6 $$y_{i} = \frac{{x_{i} - \min (x)}}{\max (x) - \min (x)}$$

where *y*_i_ is the standardized data and xi is the original data. The standardized data is shown in Table 6.

**Table 6:** Standardized data statistics.

Oxidation and acidification degree	0%	25%	50%	75%	100%
*F*_2_	0.000	0.351	0.667	0.931	1.000
*F*_*3*_	0.000	0.360	0.683	0.846	1.000

*F*_2_ refers to 2*D* fractal dimension of crystal contour; *F*_3_ refers to 3*D* fractal dimension of gray surface.

By using multiple regression analysis method^34^, the relationship between *b* (the degree of oxidation and acidification) and parameter *F*_2_ and parameter *F*_3_ is obtained, which can be expressed by Formula 4 (*R*^2^ = 0.9359).

7 $$b = 0.6039F_{2} + 0.5978F_{3} - 0.1131$$

The adjusted R^2^ = 0.9145 > 0.5 can be obtained by calculation, indicating that the data fitting degree is good. It can be seen from Formula 6 that the oxidation and acidification degree is proportional to the fractal dimension *F*_2_ of the laminated structure on the particle surface and the fractal dimension *F*_3_ of the gray surface. The standardized regression coefficient of tailings sand surface laminated structure arrangement (influencing cohesion) is 0.781, and the standardized regression coefficient of surface flatness (influencing internal friction angle) is 0.393. 0.781 is greater than 0.393, indicating that the internal friction angle is more affected by oxidation and acidification degree, which is consistent with the experimental results. The results show that the fractal dimension characteristics of tailings sand particle surface can reflect the degree of oxidation and acidification of tailings sand, and characterize the influence of oxidation and acidification on cohesion and internal friction angle.

## Conclusions

In this paper, the tailings are soaked in different concentrations of H_2_O_2_ solution to simulate the effect of oxidation and acidification of tailings with different accumulation history. The main conclusions are as follows:

1. As the degree of oxidation and acidification of tailings sand increases, the cohesion (*c*) and internal friction angle (*φ*) will decrease. The oxidation acidification of tailings sand reduces the overall mechanical properties of tailings sand. The change of mechanical properties of tailings sand under different degrees of oxidation and acidification is related to the change of mineral composition and physical properties of micro-structure of tailings sand after acidification.
2. With the increase of the degree of oxidation and acidification, the mass percentages of Ca and C in intergranular crystals show a gradual downward trend. It can be inferred that the cements between particles are CaCO_3_ (calcite) and CaMg(CO_3_)_2_. With the increase of oxidation and acidification degree, the main reason for the decrease of mechanical properties of tailings sand is that pyrite is oxidized to produce sulfuric acid, and sulfuric acid is neutralized with CaCO_3_ and CaMg(CO_3_)_2_, resulting in the loss of cementing material on the surface of tailings sand.
3. The surface structure of tailings sand is laminated structure, and mineral laminated structure is the basic structural unit of tailings sand surface under low oxidation and acidification degree. The laminated structure forms a loose matrix with weak cementation on the surface of tailings sand, and the cementing material envelops the skeleton particles of tailings. The changes of macro-cohesion and internal friction angle of tailings sand under oxidation and acidification conditions are intrinsically related to the micro-structure evolution of samples.
4. With the increase of oxidation and acidification degree of tailings sand, the 2*D* fractal dimension of the laminated structure contour of the tailings sand surface and the 3*D* fractal dimension of the gray surface gradually decrease. With the increase of oxidation and acidification degree of tailings sand, the surface roughness of tailings sand particles decreases. The standardized regression coefficient of surface laminated structure arrangement (affecting cohesion) of tailings sand is greater than that of surface flatness (affecting internal friction angle), indicating that the internal friction angle is slightly affected by oxidation and acidification.

## Data Availability

The datasets used and/or analysed during the current study available from the corresponding author on reasonable request.

## Abbreviations

SEM: Scanning electron microscope
VEDR: Viscous energy dissipation ratio
MLA: Mineral liberation analyser
EDS: Energy dispersive spectrometer
